# Residual bone fragments in tibiofibular joint and postoperative local recurrence: an analysis of 21 cases of proximal fibular giant cell tumour

**DOI:** 10.1186/s12957-018-1525-0

**Published:** 2018-11-27

**Authors:** Yun Liu, Abu Moro, Kun Wang, Xianying Huang, Changwu Wei, Kaiwei Chen, Zengming Xiao, Xinli Zhan, Haijun Tang

**Affiliations:** Department of Spine and Osteopathic Surgery, First Affiliated Hospital of Guangxi Medical University, Nanning, People’s Republic of China

**Keywords:** Giant cell tumour, Local recurrence, Tibiofibular joint, Residual bone fragment, Pathological fracture, Campanacci grade

## Abstract

**Background:**

Currently, there are no known reports on the aetiology of local giant cell tumour (GCT) recurrence in the proximal fibula following en bloc resection. We analysed 21 cases of proximal fibular GCT, focusing on the presence of residual bone in the tibiofibular joint, its causes and its impact on postoperative recurrence.

**Methods:**

We retrospectively analysed 21 cases with proximal fibular GCT occurring between 2000 and 2017.

**Results:**

There were 14 males and 7 females. The average patient age was 25.0 years. Seventeen patients were diagnosed and treated at our facility, while 4 were referred after local recurrence.

Six patients presented with residual bone fragments in the tibiofibular joint during their first month of follow-up. Patients with residual bone fragments had a higher local recurrence rate (83.3%) than those without (0%, *p* = 0.0003). Upon further analysis, patients with a preoperative Campanacci grade III tumour (*p* = 0.0055) and pathological fractures (*p* = 0.0109) were at a higher risk of exhibiting postoperative residual bone fragments.

**Conclusions:**

The presence of residual bone fragments in the tibiofibular joint was the main cause of postoperative local recurrence. The presence of residual bone fragments may be related to the preoperative Campanacci grade and pathological fractures. Therefore, close attention should be paid to postoperative follow-up examinations, and if recurrence is suspected, surgical resection should be planned.

## Background

Giant cell tumours (GCTs) of the proximal fibula are rare, accounting for only 2.7–5.2% of GCTs of the limb [1–3]. Type I en bloc resection is the preferred treatment for proximal fibular GCT as it causes less functional damage and has shown a lower postoperative local recurrence rate [3, 4]. Current reports in the literature indicate that the recurrence rate of proximal fibular GCT after type I en bloc resection ranges from 0 to 11.1% [4–7]; however, there have been no specific reports on the causes of such recurrence.

Therefore, we retrospectively analysed 21 cases of proximal fibular GCT and investigated the causes of postoperative local recurrence when present. Based on the results of the study, we propose several suggestions on how such postoperative local recurrence can be prevented or further reduced in the future.

## Methods

This retrospective study involved patients diagnosed and treated via type I en bloc resection of primary GCT in the proximal fibula at the First Affiliated Hospital of Guangxi Medical University, Nanning City, People’s Republic of China, between the years 2000 and 2017. All patients were diagnosed with GCT, and all diagnoses were confirmed histologically. All patients treated via an intralesional approach were excluded. Patients were classified according to the Campanacci et al. [8] and Jaffe [9] grading systems.

Among the 21 patients identified among the medical records, 17 underwent initial en bloc resection at our facility, while 4 were referred following local recurrence. There were 14 males and 7 females, with a mean age of 25.0 years (range, 20–49 years). The median follow-up period was 80 months, with a range of 24 to 180 months. No patients had been lost to follow-up at the time of study.

Type I en block resection for the patients treated at our facility was implemented to limit the likelihood of postoperative complications. First, the common peroneal nerve and its major branches were carefully identified and separated from the surrounding tumour mass. Depending on the local extent of the tumour, the anterior tibial artery was spared. Second, the proximal fibular with 2–3 cm of normal diaphysis and thin muscle cuff was resected. Finally, the tumour resection was completed via intra-articular resection of the tibiofibular joint by identifying and separating the tibiofibular capsule [4, 7].

## Results

### Postoperative residual bone fragments

**Fig. 1:**
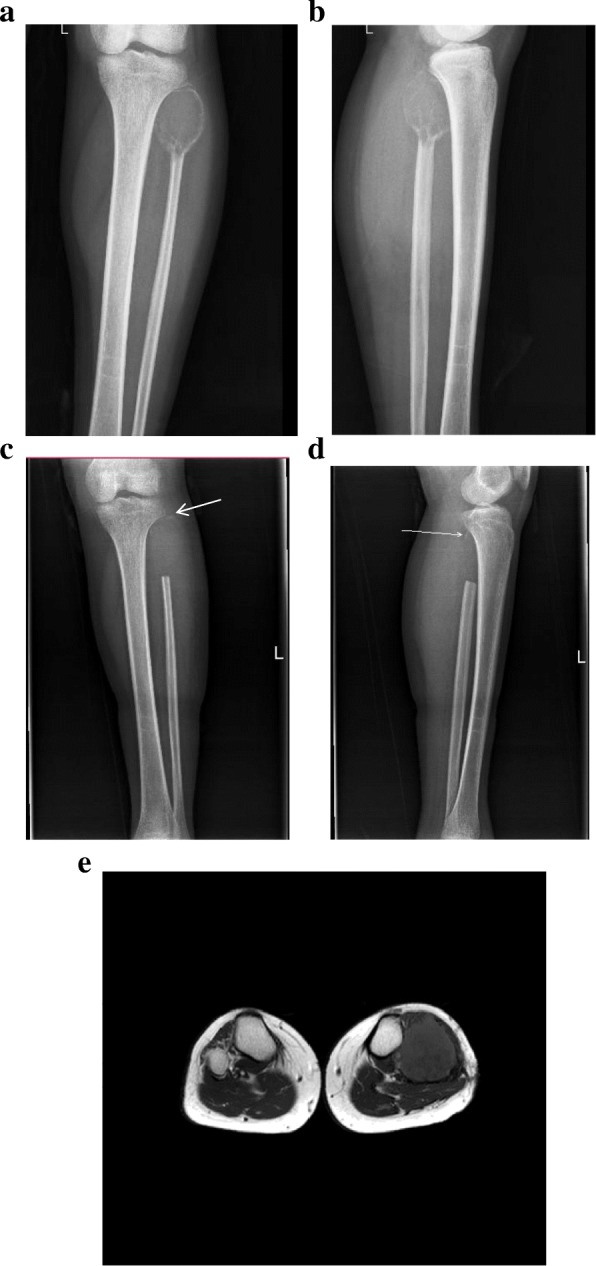
**a**, **b** Preoperative AP and lateral X-rays of the left leg of one of the patients showing the proximal fibular GCT. **c**, **d** Follow-up X-rays of the left leg of one of the patients, showing a residual bone fragment (arrows) 1 month postoperatively. **e** MRI of the same patient 8 months postoperatively, showing a soft tissue mass that had developed in the tibiofibular joint area

Analysis of the first postoperative follow-up radiographs of all 21 patients showed that 6 of the patients presented with a small high-density shadow (a residual bone fragment) in the area of the original tibiofibular joint (Fig. 1c–d). There were no high-density shadows in the humeral head area or in the fibular stump. Among the 6 patients with residual bone fragments in the tibiofibular joint, 5 developed local recurrence (83.3%). No local recurrence developed among the 15 patients without residual fragments. In terms of the postoperative local recurrence, there was a significant difference (*p* = 0.0003) between those with and without residual fragments (Table 1).

**Table 1:** Correlation analysis of the two groups (*α* = 0.05). Results are statistically significant if *p* < 0.05

	Recurrence	No recurrence	*p* value
Residual bone	5	1	0.0003
No residual bone	0	15	

### Risk factors of postoperative residual bone fragments

#### Campanacci grade

Among patients without residual fragments, 6 were Campanacci grade I, 7 were Campanacci grade II and 2 were Campanacci grade III. Among those with residual fragments, no patients were Campanacci grade I, 1 was Campanacci grade II and 5 were Campanacci grade III (Table 2). There was a significant difference between the two groups (*p* < 0.05, *p* = 0.0055).

**Table 2:** Analysis of the risk factors of residual bone fragments (*α* = 0.05). Results are statistically significant if *p* < 0.05

Category	Residual bone	No residual bone	*p*
Campanacci			0.0055
I	0	6	
II	1	7	
III	5	2	
Pathological Fractures			0.0109
No	1	12	
Yes	5	3	
Jaffe grade			0.7624
I	2	6	
II	1	4	
III	3	5	

#### Pathological fractures

There were a total of 8 patients with preoperative pathological fractures among the two groups. Five of the patients presented with postoperative residual bone fragments, while 3 did not. There were a total of 13 patients without preoperative pathological fractures; among them, 1 presented with residual bone fragment, while 12 did not (Table 2). There was a significant difference between those with and without residual bone fragments (*p* < 0.05, *p* = 0.0109).

#### Jaffe grade

Among those without residual fragments, 6 patients were Jaffe grade I, 4 were grade II and 5 were grade III. Among patients with residual fragments, 2 were Jaffe grade I, 1 was grade II and 3 were grade III (Table 2). The statistical comparison between the two groups showed no significant difference (*p* > 0.05, *p* = 0.7624).

## Discussion

GCT is a benign tumour with aggressive and recurrent characteristics. Due to the anatomical relationship between the proximal fibula and the adjacent common peroneal nerve and anterior tibial vessels, the complete resection of a proximal fibular GCT tends to be difficult [4, 7]. In our facility, type I en bloc resection is mainly used for cases of Campanacci grade III tumours, repeated recurrence and GCTs in non-weight-bearing bones, such as the fibula.

During type I en bloc resection of a proximal fibular GCT, most surgeons usually resect the tibiofibular joint intra-articularly; however, little emphasis has been placed on the effects of such a resection on postoperative local recurrence. Currently, most scholars associate the incidence of postoperative local GCT recurrence with the location of the tumour and the treatment method [1, 2]. However, there are no specific literary reports on the aetiology of the postoperative local recurrence of proximal fibular GCTs. In this study, 21 cases of proximal fibular GCT were analysed. After careful analysis of the data, we observed that 6 of the 21 patients presented with residual bone fragments in the tibiofibular joint during the postoperative follow-up examinations. Among these patients, 5 eventually developed local recurrence. Statistical analysis of those patients with and without residual bone fragments showed a significant difference between them (*p* < 0.05), suggesting that the presence of residual bone fragments in the tibiofibular joint is a major cause of postoperative local recurrence. Further analysis of the preoperative risk factors revealed that Campanacci grade III tumours and pathological fractures were important risk factors for the presence of residual bone fragments in the tibiofibular joint.

Therefore, we believe that the main reasons for the presence of residual bone fragments in the tibiofibular joint may include the following. First, if tumour growth destroys the integrity of the cortical bone, resulting in fragmentation, it becomes difficult to completely remove all fragments during surgery. Second, as proximal fibular GCTs (especially Campanacci grade III tumours) often invade the tibiofibular joint [7], it is difficult to completely remove the tumour via conventional intra-articular resection without leaving behind residual fragments. Third, because the tibiofibular joint is close to the weight-bearing knee joint and its collateral ligaments, as well as the common peroneal nerve and adjacent blood vessels, to avoid damaging these important structures, most surgeons operate too closely to the periosteum of the tumour, resulting in residual bone fragments. Finally, in patients with pathological fractures of the fibula, bone fragments from the fracture may contaminate the surrounding tissues, resulting in residual fragments.

The complete surgical resection of proximal fibular GCTs, which effectively reduces the probability of relapse, has been the focus of many recent studies [10–12]. Hu et al. [10] reported that after type I en bloc resection, there were no recurrences among the 8 cases of proximal fibular GCT they reviewed. This was because small bone fragments caused by the resection process were carefully removed. Other scholars have suggested sacrificing the peroneal nerve and tibial vessels in cases of tumours that are too large [5, 7] for complete resection to be achieved. Therefore, we suggest that during and after type I en bloc resection of the proximal fibula, the following points should be taken into account. (1) For Campanacci grade III tumours, perhaps it is advisable to perform extra-articular resection of the tibiofibular joint, as the tumour is more likely to invade the tibiofibular joint. (2) The operation space should be sufficiently large to adequately expose the tumour mass, collateral ligaments, common peroneal nerve and tibial vessels. (3) During the operation, the knee joint should be in a relaxed state to reduce tension in the muscles and tendons around the fibula. This approach may prevent the unintentional dislodgement of bone fragments due to tension in a muscle or tendon. (4) It is very important to carefully examine the postoperative follow-up radiographs of patients. Once residual bone fragments are observed, close attention should be paid to subsequent follow-up examinations, and if a recurrence is suspected, surgical resection should be planned as soon as possible.

The limitations of this study are that the sample size is relatively small, and not all patients were diagnosed and treated at our facility. Since different surgeons have different experience levels and operative approaches, the conclusions of this study need to be further confirmed in multicentre studies.

## Conclusions

Preoperative Campanacci grade III tumours and pathological fractures are the main risk factors for the presence of residual bone fragments after the en bloc resection of proximal fibular GCTs. Such residual bone fragments may result in postoperative local recurrence. Therefore, it is very important to perform extra-articular resection in high-risk patients. Additionally, very close attention should be paid to the follow-up radiographs of such patients, and if residual fragments are observed, the next course of treatment should be planned.
